# α_1_-Microglobulin Binds Illuminated Flavins and Has a Protective Effect Against Sublethal Riboflavin-Induced Damage in Retinal Epithelial Cells

**DOI:** 10.3389/fphys.2020.00295

**Published:** 2020-04-02

**Authors:** Jesper Bergwik, Bo Åkerström

**Affiliations:** Division of Infection Medicine, Department of Clinical Sciences, Lund University, Lund, Sweden

**Keywords:** α_1_-microglobulin, riboflavin, FAD, oxidative stress, retina, skin, antioxidant, vitamin B2

## Abstract

Riboflavin (vitamin B2) is an important constituent of the prosthetic groups flavin adenine dinucleotide (FAD) and flavin mononucleotide (FMN), which are utilized as electron-carriers in energy metabolism. Excitation by UV-light leads to the generation of riboflavin radicals and reactive oxygen species (ROS), which can oxidize a wide range of biomolecules. The human protein α_1_-microglobulin (A1M) is a reductase and a radical scavenger, which can protect cells and matrix against oxidative damage. Here, we provide evidence of a molecular interaction between illuminated riboflavin and A1M, similar to the radical scavenging reactions previously seen between A1M and other organic radicals. Binding between riboflavin and A1M was demonstrated by gel migration shift, UV-absorbance and fluorescence spectrum analysis. The reaction between A1M and UV-light illuminated riboflavin involved covalent modification of A1M and proteolytic release of an N-terminal part of the protein. Furthermore, A1M also inhibited the ROS-induced photoreduction reaction of riboflavin, in a reaction involving the free thiol group in position C34. Finally, the results show a protective effect of A1M, analyzed by gene expression rates of stress genes, against sublethal damage in retinal epithelial cells in culture. Together, our results suggest a new role of A1M as a scavenger of riboflavin radicals and ROS produced during illumination of riboflavin.

## Introduction

Riboflavin (7,8-dimethyl-10-ribityl-isoalloxazine), which is generally referred to as vitamin B2, is a water-soluble vitamin that was discovered in 1872. Milk, eggs, meat, and leafy vegetables have a high content of riboflavin, which makes them a main source of the vitamin. Animals cannot synthesize riboflavin, and it is therefore an essential vitamin. The most vital role of riboflavin is as a constituent of the prosthetic groups flavin adenine dinucleotide (FAD) and flavin mononucleotide (FMN), which are important cofactors in energy metabolism. Riboflavin is mostly present as part of FAD and FMN, but it also exists in its free form (Edwards, 2006). Riboflavin, FAD and FMN can participate in one- and two-electron transfer processes, which means that the molecules can exist in different redox states; oxidized, one-electron reduced, and two-electron reduced. Flavins can thus participate in transfer of single electrons, hydrogen atoms and hydride ions. These are the major reasons for the ubiquity of flavin enzymes in the human body.

When riboflavin is illuminated it rapidly undergoes a process referred to as photo-degradation, which generates different reactive oxygen species (ROS). During the photo-degradation, riboflavin is broken down into several different molecules, where lumichrome, formylmethylflavin and lumiflavin are the most abundant at physiological pH (Ahmad and Vaid, 2006). The illumination results in a spin-allowed transition to a highly fluorescent short-lived singlet excited state (^1^Rib^∗^). Through an intersystem crossing, a more long-lived triplet-excited state (^3^Rib^∗^) is generated. ^3^Rib^∗^ is a bi-radical and a powerful oxidant, which can directly oxidize a wide range of biomolecules (Heelis, 1982). The direct interaction between ^3^Rib^∗^ and biomolecules is known as the type I mechanism (Rochette et al., 2000). The type I mechanism leads to the generation of superoxide anion radicals (O_2_^*–^). The superoxide anion radical can subsequently form H_2_O_2_ and hydroxyl radicals (^∗^OH) (Cardoso et al., 2012). ^3^Rib^∗^ may also transfer the excitation energy to ground-state oxygen to yield singlet oxygen, which is termed the type II mechanism (Rochette et al., 2000).

Riboflavin, FAD and FMN are abundant in organs with a high metabolic activity, but they are also present in light-exposed tissue, such as the skin and eyes (Cardoso et al., 2012). The illumination in these tissues will lead to the generation of the previously described radicals, and these can react with DNA, proteins and lipids, which can lead to cell death, mutations, chromosomal aberrations and carcinogenesis (Khan et al., 2010). For example, oxygen radicals have been reported to be key mediators of DNA damage during the development of cancer (Valko et al., 2004; Lau et al., 2008). Other publications also show that UVA-induced mutations in the DNA of fibroblasts are increased several fold when riboflavin acts as a photosensitizer (Besaratinia et al., 2007). Furthermore, oxidative stress has been shown to be one of the primary causes of several different eye diseases, such as cataract, glaucoma, age-related macular degeneration (AMD) and rhegmatogenous retinal detachment (RRD) (Alge et al., 2002; Izzotti et al., 2006; Ho et al., 2010; Roh et al., 2011). There is obviously a need for a protection mechanism against flavin-generated radicals in both the skin and the eye.

The lipocalin α_1_-microglobulin (A1M) is a 26 kDa plasma and tissue protein mainly synthesized in the liver, but also in smaller amounts in peripheral organs. A1M is secreted into the blood stream, from the liver, and found in blood as complexes with IgA, albumin and pro-thrombin (∼1 μM) and in its free form (∼1 μM) (DeMars et al., 1989; Berggård et al., 1997). From the bloodstream it is further transported into the extravascular compartments (Larsson et al., 2001). Several different biochemical properties have been described for A1M. Firstly, a radical trapping function, where A1M covalently binds small organic radicals to lysyl and tyrosyl side-chains, has been demonstrated (Åkerström et al., 2007). Secondly, a reductase ability dependent on a free cysteine in position 34 (Cys34) has been described, where NADH, NADPH and ascorbate could function as electron donating cofactors (Allhorn et al., 2005). Thirdly, A1M was found to bind heme (Larsson et al., 2004; Siebel et al., 2012; Karnaukhova et al., 2014; Rutardottir et al., 2016) and a truncated form (t-A1M) showed a heme-degrading function (Allhorn et al., 2002). Additionally, ROS induce up-regulation of gene expression of A1M in different cell and tissue types, such as skin keratinocytes and retina explants from rat and pig, *in vitro* (Olsson et al., 2007, 2011; Cederlund et al., 2013; Åkerström et al., 2017). Increased gene expression of A1M was also found *in vivo* during conditions with excessive free hemoglobin (Olsson et al., 2010a, 2012; Anderson et al., 2011; Gram et al., 2015). A1M has also been shown to protect bystander cells in α particle-irradiated cell cultures, where the damage is caused by oxidative stress through the generation of free radicals, ROS and oxidants (Olsson et al., 2010b; Rutardottir et al., 2013). More recently, in a mouse radiation model, A1M was shown to protect the kidneys from radiation damage (Kristiansson et al., 2018). The protective properties of A1M are physiologically relevant in cells due to uptake of A1M into cells and binding to mitochondria (Olsson et al., 2013) and in matrix due to binding to collagen fibrils (Olsson et al., 2011).

In this paper we hypothesize that A1M can bind to riboflavin and react with ROS generated during illumination of flavins, and thereby protect cells and tissues from oxidative damage. We have investigated the biochemical interactions between flavins and A1M and studied the protective effects of A1M against illuminated riboflavin-induced stress in retinal epithelial cell cultures.

## Materials and Methods

### Proteins and Reagents

Recombinant human A1M with an N-terminal His-tag, was expressed in *E. coli*, purified and refolded as previously described (Kwasek et al., 2007). A recombinant mutated form of A1M, A1M-C34S, was constructed, expressed and purified as described (Rutardottir et al., 2013). Ovalbumin (OVA), human serum albumin (HSA), riboflavin and FAD were bought from Sigma-Aldrich (St. Louis, MO, United States).

### UV Absorbance Scanning

Stock solutions were made by dissolving riboflavin and FAD in dimethyl sulfoxide (DMSO) to a concentration of 50 mM. The samples were prepared by adding riboflavin (1.22 mM), FAD (1.22 mM) and/or A1M (1.5 mg/ml), C34S (1.5 mg/ml), OVA (1.5 mg/ml) or HSA (1.5 mg/ml) to Eppendorf tubes followed by illumination with a fluorescent lamp (1200 lumen) at a distance of 0.2 m for 2 h. After the incubation, the proteins were separated from the riboflavin/FAD-mixture through desalting on a G25 column. An absorbance spectrum between 240 and 700 nm was measured for each sample using a DU800 UV/Vis spectrophotometer (Beckman Coulter Diagnostics, CA, United States).

### Fluorescence Spectra

The riboflavin stock solution was prepared as described above. The fluorescence measurements of A1M was measured using a Jasco J-810 spectrofluorimeter (equipped with an FMO-427 monochromator). A1M (2.2 μM) in 20 mM Tris–HCl, 0.15 M NaCl, pH 8.0, was added to a 100 μl quartz cuvette (Hellma Precision Cell, Type no. 105.251-QS, Light-path length 3 mm in both excitation and emission modes). Tryptophan fluorescence was measured with excitation at 290 nm and fluorescence emission detection at 320–400 nm with slits set at 5 nm bandwidth. The following concentrations of riboflavin was added and measured: 0.625, 1.25, 2.5, 5, 10 and 20 μM. All solutions were kept dark to avoid photodegradation, except during the actual fluorescence measurements. DMSO was also added to the A1M solution alone to verify that it did not affect the emission spectra.

### SDS-PAGE and Western Blotting

The riboflavin and FAD stock solutions were made as described above. The samples were prepared through addition of riboflavin or FAD to Eppendorf tubes to a final concentration of 40, 200 or 1000 μM. Recombinant A1M was then added to the tubes to a final concentration of 100 μM. The tubes were illuminated with a fluorescent lamp (1200 lumen) at a distance of 0.2 m for 1 h in RT. The non-illuminated tubes were wrapped in tinfoil. SDS-PAGE was performed as described (Laemmli, 1970) with 12% TGX Stain-free Gels (Bio-Rad Laboratories, Hercules, CA, United States). The samples were reduced through addition of β-mercaptoethanol (5%) to the sample buffer followed by boiling for 1 min before applying the samples to the gel. Electrophoresis was performed at 200 V for approximately 40 min. The gels were stained with Coomassie (Bio-Safe^TM^ Coomassie G-250 stain, Bio-Rad) or transferred to a polyvinylidene fluoride (PVDF) membrane (Transblot^®^ Turbo, Bio-Rad). Rabbit anti-LIPR (leucine-isoleucine-proline-arginine; the C-terminal tetrapeptide of A1M), prepared in-house by immunization with KLH-conjugated LIPR (Allhorn et al., 2002) (Agrisera AB, Vännäs, Sweden), and mouse anti-Histidine-tag (AbD Serotec, Kidlington, United Kingdom), were used as the primary antibodies and incubated overnight at 4°C. Alexa Fluor 647 goat anti-mouse IgG (Thermo Fisher Scientific, Waltham, MA, United States) and Alexa Fluor 647 goat anti-rabbit (Thermo Fisher Scientific) were used as the secondary antibodies, respectively, and incubated at RT for 1 h. The membranes and gels were analyzed using a ChemiDoc^TM^ MP System (Bio-Rad).

### Cell Culturing

Retinal pigmented epithelium cells (ARPE19, ATCC, Manassas, VA, United States) were grown in DMEM: F-12 medium supplemented with 10% FBS and 1% PEST (Thermo Fisher Scientific) in T75 flasks. The cells were seeded in 12-well plates and grown until confluent. The cells were incubated with A1M (1, 5 or 10 μM) and/or riboflavin (200 μM) followed by 15 min of illumination with a fluorescent lamp (1200 lumen) at a distance of 0.2 m. After illumination the plates were incubated in the dark at 37°C for 4 h. No controls could be performed in complete darkness since cell handling in cultivation hoods had to be done under normal light. The cell medium was aspirated and stored in tubes. TRIzol^®^ was added to the wells followed by scraping of the surface using a Falcon^®^ cell scraper (Corning Incorporated, Corning, NY, United States) to release the cells.

### Real-Time PCR

RNA was extracted from the cell extract, collected with TRIzol^®^ (Thermo Fisher Scientific), using a Quick-RNA^TM^ MiniPrep kit (Zymo Research, Irvine, CA, United States). The RNA concentrations were measured using a NanoDrop spectrophotometer ND1000 (Saveen Werner AB, Malmö, Sweden). cDNA (1 μg) was prepared using an iScript^TM^ cDNA Synthesis Kit (Bio-Rad). RT-PCR was used to analyze the mRNA levels of the house-keeping gene glyceraldehyde-3-phosphate-dehydrogenase (*GAPDH*), the oxidative stress marker heme oxygenase-1 (*HMOX1*) and the cell cycle regulator *p21*. All primers were obtained from Eurofins Scientific (Brussels, Belgium). The expression was analyzed with iQ SYBR Green Supermix (Bio-Rad). Raw data was obtained as cycle threshold values (Ct-values). These were normalized to the Ct-values of the house-keeping gene *GAPDH*, and thus expressed as ΔCt-values. The ΔCt-values of the treated cells were compared to the ΔCt-values of the control cells generating ΔΔCt-values.

### Statistical Analysis

In the RT-PCR data, values are given as mean ± SD with 3 samples per condition. Statistical analysis was performed with a one-way ANOVA with Sidak multiple comparison test using Prism 8, version 8.0.2 (159) (GraphPad Software, La Jolla, CA, United States).

## Results

### Binding of Riboflavin to A1M

**FIGURE 1:**
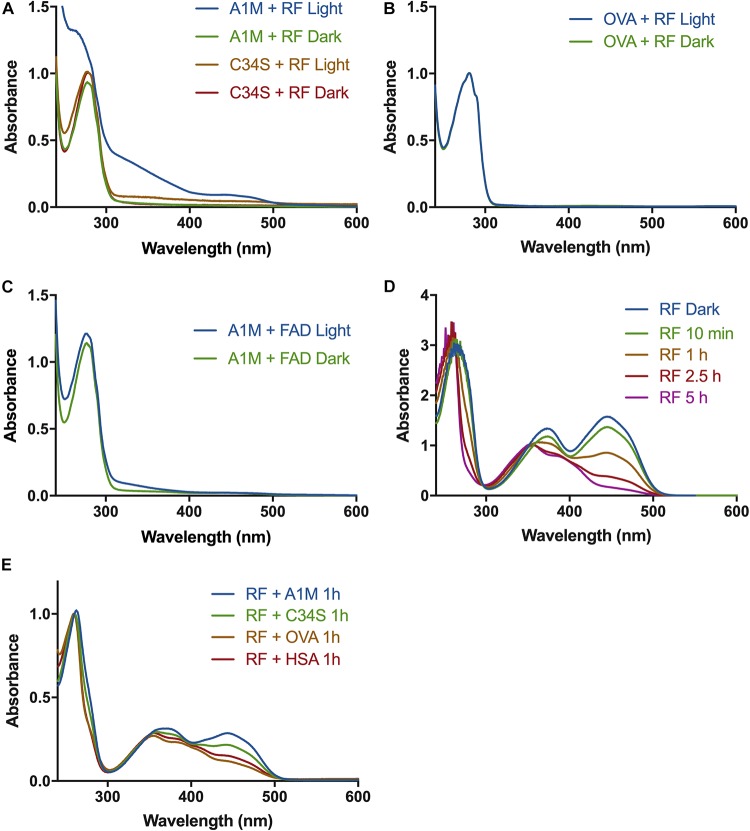
Absorbance spectra of A1M and riboflavin after exposure to light. **(A)** A1M (1.5 mg/ml) or A1M-C34S (1.5 mg/ml) and riboflavin (1.22 mM) were illuminated with a fluorescent lamp (1200 lumen) at a distance of 0.2 m, or kept in darkness, for 2 h. The proteins were separated from the riboflavin and a UV-absorbance spectrum of the protein fraction was measured from 240–700 nm. **(B)** Ovalbumin (1.5 mg/ml) and riboflavin (1.22 mM) were illuminated, or kept in darkness, and measured as described in **(A)**. **(C)** A1M (1.5 mg/ml) and FAD (1.22 mM) were illuminated, separated and measured as described in **(A)**. **(D)** Riboflavin (20 μM) was illuminated as described in **(A)** and a spectrum was measured at the indicated time points. **(E)** Riboflavin (1.22 mM) was incubated with A1M, C34S, OVA, or HSA and illuminated for 2 h, separated from the proteins and measured as described in **(A)**.

A1M was incubated with riboflavin under illumination, followed by separation of protein and free riboflavin by desalting on a G25 column. The fractions containing A1M were analyzed by reading the absorbance spectrum between 240 and 700 nm. Increased absorbance of A1M was seen after reacting with the illuminated riboflavin. Peaks/shoulders in the spectrum of A1M were seen at approximately 250, 360, and 450 nm, indicating a binding of the riboflavin molecule to A1M (Figure 1A). A similar binding to riboflavin, but to less extent, was seen with the mutated A1M-variant A1M-C34S, where the redox-active free thiol group at pos. 34 is replaced by a serine residue. No change in the absorbance spectra could be seen with A1M alone, when A1M was incubated with riboflavin in darkness, or when incubating ovalbumin (Figure 1B) or HSA (not shown) with illuminated riboflavin under the same conditions. When incubating A1M with FAD, which has a riboflavin molecule as a prosthetic group, under the same conditions there was a similar, but smaller, increase in peaks/shoulders (Figure 1C).

### The Effect of A1M on the Photo-Degradation of Riboflavin

It has previously been shown that the antioxidants Trolox (vitamin E analog) and ascorbic acid (de La Rochette et al., 2000; Jung and Min, 2009) can inhibit the photo-degradation process of riboflavin. We therefore examined if A1M has a similar effect by mixing protein and riboflavin, followed by illumination, incubation and separation by gel filtration. During the photo-degradation of riboflavin the 360 and 450 nm peaks decreased over time (Figure 1D). When adding A1M the photo-degradation was slowed down, suggesting that the degradation was partly inhibited by A1M (Figure 1E). The partial inhibition of riboflavin photoreduction seen by A1M-C34S was weaker than by non-mutated A1M, suggesting that the C34 free thiol group of A1M is involved in the reaction (Figure 1E). No inhibition was seen by control proteins ovalbumin and HSA (Figure 1E). The photo-degradation of FAD was much slower and did not allow any analysis of the inhibition by A1M (not shown).

### Tryptophan Fluorescence Is Quenched When A1M Binds Riboflavin

**FIGURE 2:**
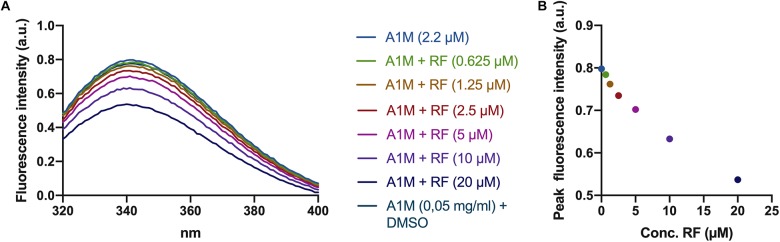
Riboflavin-induced tryptophan fluorescence quenching in A1M. **(A)** Fluorescence spectra of A1M between 320 and 400 nm. Increasing amounts of riboflavin (0.625, 1.25, 2.5, 5, 10, and 20 μM) was added to a solution of A1M (2.2 μM = 0.05 mg/ml) followed by fluorescence measurements using a Jasco J-810 spectropolarimeter. **(B)** Peak fluorescence intensity of the same concentrations of riboflavin and A1M as in **(A)**.

A tryptophan residue is located at the bottom of the A1M pocket. The fluorescence of the tryptophan has previously been shown to be quenched when A1M binds a ligand in its pocket (Rutardottir et al., 2016). When A1M was incubated with riboflavin, there was a dose dependent decrease of the fluorescence peak at 340 nm (Figures 2A,B), indicating a binding of riboflavin in the pocket. No decrease in fluorescence was seen when adding equivalent amounts of DMSO.

### Molecular Changes of A1M

**FIGURE 3:**
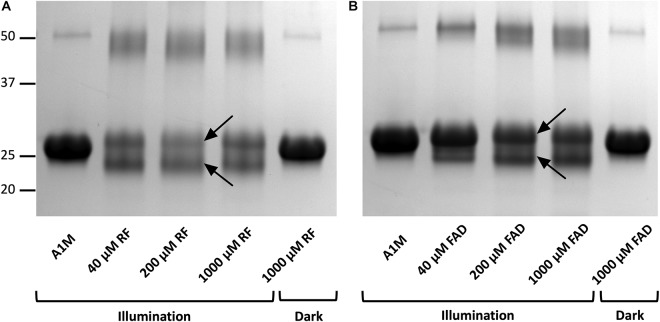
Gel electrophoresis after incubation of A1M with **(A)** riboflavin and **(B)** FAD during illumination or in darkness. Recombinant A1M (10 μM) was incubated with buffer, 40, 200, and 1000 μM riboflavin **(A)** or FAD **(B)**. The samples were illuminated with a fluorescent lamp (1200 lumen) at a distance of 0.2 m or kept in darkness for 1 h. The gels were stained with Coomassie Brilliant Blue and analyzed using a ChemiDoc^TM^ MP System (Bio-Rad). Upper and lower arrows indicate the full length modified A1M and the cleavage product, respectively, after the reactions with riboflavin or FAD.

When A1M was incubated with illuminated riboflavin (Figure 3A) or FAD (Figure 3B) and analyzed by SDS-PAGE, two molecular changes of the protein could be observed. First, an increased apparent molecular mass of the main band of A1M was seen at ∼26 kDa, compared to A1M without riboflavin or riboflavin in darkness, both at ∼25 kDa. Secondly, A1M was cleaved into two separate bands: the major band was apparently intact and a new band of ∼22 kDa in size was formed (Figures 3A,B). When A1M was incubated with riboflavin or FAD, without illumination, no change in size or cleavage was seen. The results also show that A1M alone was not affected by the illumination. Intensity measurements of the two bands formed after incubation with 40 μM riboflavin or FAD, showed a slightly higher cleaving rate for riboflavin compared to FAD, 51%, respectively, 45%. A dimeric form of A1M (∼50 kDa) was seen in all samples. More dimers were generated in the illuminated samples and they had a lower molecular weight, indicating that the dimer consists of two cleaved A1M molecules. Similar results were obtained using non-reducing conditions.

### Localization of A1M Cleavage

**FIGURE 4:**
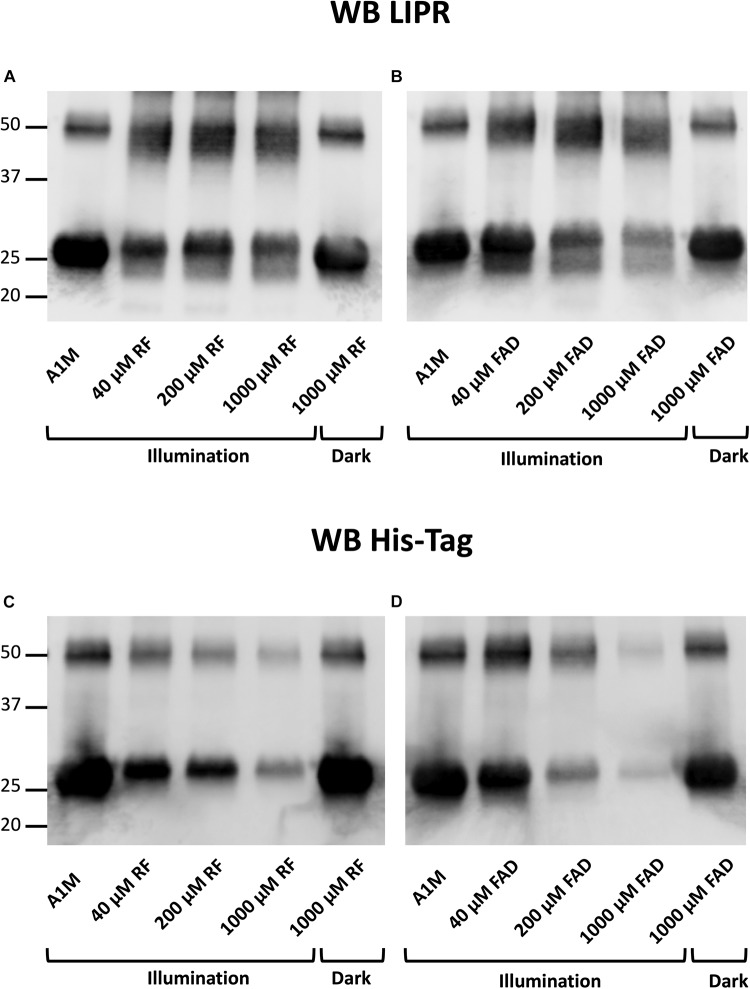
Western blotting after incubation of A1M with **(A,C)** riboflavin and **(B,D)** FAD during illumination or in darkness. Recombinant A1M 10 μM was incubated with buffer, 40, 200 or 1000 μM riboflavin **(A)** or FAD **(B)**. The samples were illuminated with a fluorescent lamp (1200 lumen) at a distance of 0.2 m or kept in darkness for 1 h. **(A,B)** were blotted with antibodies against the C-terminal peptide LIPR. **(C,D)** were blotted with antibodies against the N-terminal His-tag. The membranes were analyzed using a ChemiDoc^TM^ MP System (Bio-Rad).

To analyze where the A1M molecule was cleaved during the incubation with the illuminated riboflavin or FAD, Western Blotting of the A1M-products was performed, using antibodies against the C-terminal peptide LIPR (Allhorn et al., 2002) and the N-terminal His-tag. The results show that the C-terminal peptide LIPR was present in the cleaved A1M-band (Figures 4A,B), suggesting that the C-terminal part of A1M was intact. Furthermore, when blotting against the N-terminal His-tag, the smaller band was not seen (Figures 4C,D). The presence of the C-terminus and the disappearance of the N-terminus were seen when incubating both with riboflavin and with FAD. There was a dose dependent decrease in the staining intensity of the bands with anti His-tag antibodies for both riboflavin and FAD. This occurred for both the monomer and the dimer of A1M. These results indicate that the N-terminal end of the A1M molecule is partially cleaved off when it is incubated with riboflavin or FAD.

### A1M Protects Cells From Riboflavin-Induced Oxidative Stress

**FIGURE 5:**
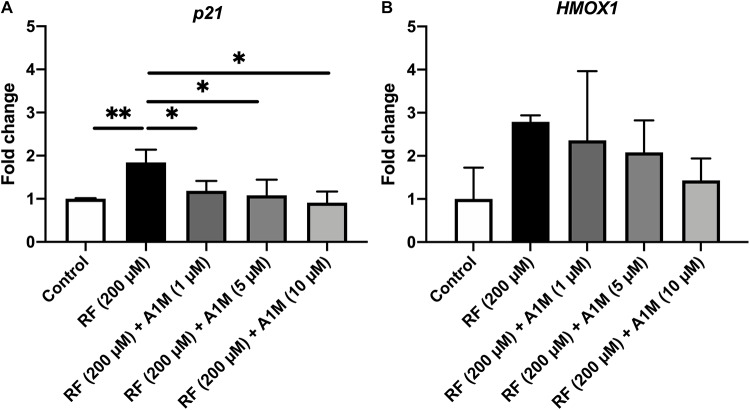
The effects of illuminated riboflavin and A1M on the relative mRNA levels of **(A)** *p21* and **(B)** *HMOX1* in ARPE19 cells. The cells were treated with riboflavin with or without A1M and illuminated with a fluorescent lamp (1200 lumen) at a distance of 0.2 m. The mRNA expression was measured with iQ SYBR Green Supermix (Bio-Rad). Primers were obtained from Eurofins Scientific. Raw data were obtained as Ct-values, which were normalized to the Ct-values of the house-keeping gene *GAPDH* (= ΔCt). The expression is relative to the control samples (= ΔΔ^*Ct*^) and is shown as fold change (2^–ΔΔ*Ct*^). Statistical comparisons between samples were performed with one-way ANOVA with Sidak multiple comparison test. *p < 0.05, **p < 0.01.

When adding riboflavin to cultures of ARPE19 cells and illuminating them, there was a significant increase in mRNA levels of the cell cycle regulator *p21* (*p* < 0.01) and a tendency to an increase of the oxidative stress marker heme oxygenase 1 (*HMOX1)* (Figures 5A,B). When also adding A1M to the cultures, prior to illumination, there was a significant decrease in the mRNA levels of *p21* (1 μM, *p* < 0.05; 10 μM, *p* < 0.01) and a trend toward a decrease in the mRNA of HMOX1. The results also demonstrated a dose dependent protective effect of A1M, where a higher concentration gives further protection.

## Discussion

Riboflavin, or vitamin B2, is present in light-exposed parts of the human body (Cardoso et al., 2012), which render these tissues exposed to oxidative stress from riboflavin-derived radicals and ROS generated by illumination of the vitamin. The focus of this investigation is A1M, a potential endogenous protective mechanism against harmful effects of illuminated riboflavin in light exposed tissues. Previously it has been shown that A1M, a human reductase and radical-binding protein, is transported from the blood vessels into the extravascular compartments of the skin, where it is also expressed by the keratinocytes during ROS exposure (Olsson et al., 2011). A1M is also found in the eye, expressed by retina, and its levels in the vitreous are elevated in patients with eye diseases connected to oxidative stress (Cederlund et al., 2013). The co-localization of A1M and riboflavin in skin and eye suggests a possible physiological protective effect of A1M against riboflavin radicals in these tissues.

The results suggest that illuminated riboflavin and FAD are bound specifically to A1M. When A1M is incubated with illuminated riboflavin, three peaks at approximately 250, 360, and 450 nm appear (Figure 1A). These peaks, however, are not present in the samples with A1M only, ovalbumin or non-illuminated riboflavin. Additionally, the tryptophan located at the bottom of the A1M pocket (W25), which has previously been shown to be quenched when A1M binds a ligand, is quenched in a dose dependent manner with an increasing concentration of riboflavin, further establishing the binding (Figures 2A,B).

The A1M-riboflavin complex was slightly larger than unmodified A1M on SDS-PAGE suggesting a covalent binding. Since the shifts in absorbance and SDS-PAGE is only seen when riboflavin is illuminated, the binding appears to be specific for radicals formed during the photo-degradation. We further analyzed the structural changes and found that A1M also is cleaved, generating a second band (Figures 3A,B). Unmodified A1M had an apparent size of 25 kDa, the A1M-riboflavin band had a size of 26 kDa and the newly formed A1M-fragment was approximately 4 kDa smaller. A similar formation of an A1M-fragment was also seen when incubating A1M with the synthetic radical ABTS (Åkerström et al., 2007). This suggests that the reaction between A1M and riboflavin is similar to the reaction between A1M and ABTS and may represent a general reaction mechanism of A1M upon radical binding. A dimer, around 50 kDa, was also formed during the incubation in all samples. The results shown in Figure 3 suggest that the cleaved form of A1M also can dimerize in the presence of illuminated riboflavin. SDS-PAGE under non-reducing conditions yielded an identical pattern, suggesting that the cleavage peptide or the dimers were not dependent on disulfide bonds. The reaction between A1M and riboflavin was very similar to that between A1M and FAD. However, cleaved A1M (lower band) was formed to a larger extent by riboflavin (51%) compared to FAD (45%), indicating that riboflavin is more reactive than FAD.

We further investigated the cleaved A1M band by Western Blot with antibodies against the C-terminal peptide LIPR and the N-terminal His-tag. The results showed that the A1M-fragment generated by incubating A1M with illuminated riboflavin or FAD lacked the N-terminal His-tag but had an intact C-terminus (Figure 4). This suggests that A1M is cleaved at a site closer to the N-terminus than to the C-terminus when it is incubated with illuminated riboflavin and FAD. A1M was also shown to be cleaved at the N-terminal when reacting with the radical ABTS (Åkerström et al., 2007) further supporting the hypothesis that the riboflavin radical binding mechanism is similar to the ABTS binding mechanism.

The generation of ROS by illuminated flavins is thought to enhance the rate of photo-degradation in a positive feedback loop (Huang et al., 2004). We therefore hypothesized that A1M could decrease the rate of photo-degradation by reacting with the generated ROS (Åkerström et al., 2007). The results support that A1M indeed slows the photo-degradation of riboflavin (Figure 1D). The mutated A1M-variant, A1M-C34S, lacking the redox active free thiol group at pos. 34, showed less inhibitory effect, although remaining activity was clearly seen (Figure 1D). The C34 residue was previously shown to be essential for the reductase activity of A1M (Allhorn et al., 2005) and the A1M-C34S mutant displayed a reduced radical-binding activity (Åkerström et al., 2007). Since this residue also seems to be involved in inhibition of the photo-reduction of riboflavin, the results thus support the hypothesis that A1M has the capacity to bind the riboflavin-generated ROS. However, the C34 thiol group is apparently not an absolute requisite for the binding of riboflavin to the A1M protein (Figure 1A).

To test the hypothesis that A1M, as a radical scavenger, may inhibit biological toxic effects of riboflavin, we investigated if A1M could protect retinal epithelial cells from riboflavin radicals generated during illumination. When measuring the mRNA levels of the cell cycle regulator *p21*, a significant dose dependent decrease in expression was seen with A1M treatment (Figure 5A). A similar trend was found for the oxidative stress marker *HMOX1*, but statistically significant difference was not achieved (Figure 5B). These results suggest that A1M has a protective effect against intracellular sublethal damage. Internalization of A1M in skin keratinocytes has previously been reported (Olsson et al., 2011), further supporting a hypothetical, potential protection mechanism against riboflavin radicals formed intracellularly.

Together, our results suggest a role of A1M as a scavenger of riboflavin radicals and ROS produced during illumination. A potential mechanism of A1M may be to bind the riboflavin and react with the radicals formed by downstream reactions in the skin and eyes during illumination and thereby preventing oxidative stress-related diseases.

## Data Availability Statement

The datasets generated for this study are available on request to the corresponding author.

## Author Contributions

JB and BÅ contributed to the design of the study and performed the experiments. JB wrote the manuscript. Both authors contributed to manuscript revision and approved the submitted version.

## Conflict of Interest

The authors declare that this study received funding from A1M Pharma. The funder was not involved in the study design, collection, analysis, interpretation of data, the writing of this article or the decision to submit it for publication. BÅ owns A1M Pharma shares.

The remaining author declares that the research was conducted in the absence of any commercial or financial relationships that could be construed as a potential conflict of interest.
